# Effects of SARS-CoV-2 infection on hypothyroidism and subclinical hypothyroidism: a meta-analysis

**DOI:** 10.3389/fendo.2023.1291774

**Published:** 2023-12-04

**Authors:** Jiaqi Wei, Fenghua Zhang

**Affiliations:** ^1^College of Medical Technology, Shanghai University of Medicine & Health Sciences, Shanghai, China; ^2^Department of Laboratory Medicine, Shanghai University of Medicine & Health Sciences Affiliated Zhoupu Hospital, Shanghai, China

**Keywords:** SARS-CoV-2, COVID-19, hypothyroidism, subclinical hypothyroidism, meta-analysis

## Abstract

**Background:**

In recent years, the outbreak of COVID-19 caused by SARS-CoV-2 has been witnessed globally. However, the impact of SARS-CoV-2 infection on thyroid dysfunction and subclinical thyroid dysfunction remains unclear. Therefore, this meta-analysis aimed to assess the effects of SARS-CoV-2 infection on thyroid dysfunction and its relationship with the severity of COVID-19.

**Methods:**

We systematically searched databases including PubMed, Willey Library, Embase, Web of Science, CNKI, Wanfang, and VIP. We focused on randomized controlled trials, case-control studies, and cohort studies published between December 2019 and August 2023, examining the association between SARS-CoV-2 infection and hypothyroidism, with a specific emphasis on the severity of the infection. The quality of the research was assessed using the Newcastle-Ottawa Scale (NOS), while statistical analysis was conducted using the meta and metafor packages in R 4.2.1 software.

**Results:**

For the meta-analysis, a total of eight articles were identified based on strict inclusion and exclusion criteria. For the association between SARS-CoV-2 infection and hypothyroidism, three studies (266 samples) comparing TSH levels of COVID-19 and control groups showed no difference in TSH levels [SMD=-0.04,95%CI(-1.22,1.15),*P=0.95*]. Additionally, two studies examining TT3 (a sample of 176 cases) and two studies examining TT4 (a sample of 176 cases) also showed no difference in TT3 and TT4 between the COVID-19 group and the control group, respectively. However, when evaluating the severity of COVID-19, six studies (565 samples) showed that TSH in the severe group was significantly lower than in the mild group [SMD = -0.55, 95% CI (-0.96, -0.14)], while FT3 was also lower in the severe group [SMD = -0.96, 95% CI (-1.24, -0.67)]. No noticeable differences were observed between the severe and mild groups in their TT3, FT4, and TT4 levels.

**Conclusion:**

SARS-CoV-2 infection may have detrimental effects on thyroid function in individuals with severe symptoms. More research is needed to confirm and explore this relationship.

**Systematic Review Registration:**

https://www.crd.york.ac.uk/PROSPERO, identifier CRD42023486042.

## 1 Introduction

Since the initial outbreak of SARS-CoV-2 coronavirus at the end of 2019, the world has been grappling with this unprecedented health crisis, leading to hundreds of millions of infections and innumerable fatalities (1, 2). Early research and clinical observations have focused on how the virus causes direct damage to the respiratory system, especially the lungs (3). However, as time progressed, extensive clinical and laboratory studies have revealed the multifaceted effects of the virus on other organs and physiological systems, with the involvement of the endocrine system receiving particular attention from researchers (4, 5). The thyroid gland serves an essential role in many of the basic physiological processes that occur in the body, such as regulating body temperature, controlling basal metabolic rate, and many other processes related to metabolism, growth and development (6). Moreover, the synthesis, storage, and release of thyroid hormones are also critical factors in maintaining overall homeostasis (7). Therefore, any external agents, particularly novel viruses like SARS-CoV-2 (8, 9), that might impede thyroid function necessitate meticulous and systematic investigation.

Recently, several studies have sought to examine the potential associations between SARS-CoV-2 and thyroid function. Distinct biochemical anomalies in thyroid function have been documented in specific cases, predominantly in the nascent phase of COVID-19 infection or during its more severe manifestations (10–12). For example, certain research noted that a subset of COVID-19 patients had biochemical changes similar to subclinical hypothyroidism after virus invasion, especially the increase of thyroid stimulating hormone (TSH) (10, 11). Subclinical hypothyroidism is defined as elevated serum TSH levels with normal serum thyroid hormones (T4 and T3) in patients with no symptoms of hypothyroidism or only mild symptoms of hypothyroidism. In addition, there are studies suggesting that COVD-19 may trigger an inflammatory response in the thyroid gland, leading to transient hypothyroidism (13). Nevertheless, these studies do not always converge on a shared conclusion (10–16). The inconsistency may be due to a variety of factors, such as the differences in biochemical detection methods used, the population characteristics of the subjects, the geographical environment, and the specific genotypes or variants of SARS-CoV-2 involved. Therefore, any interpretation of these preliminary findings needs to be cautious and take into account these potential confounding factors.

Given that recent studies have drawn divergent conclusions on the association between COVID-19 and thyroid function, a comprehensive and consistent analysis is needed. This can be best captured by a meta-analysis, which systematically integrates studies on thyroid dysfunction associated with SARS-CoV-2 infection, with particular attention to the correlation between COVID severity and hypothyroidism. Through this methodology, we hoped to achieve a clearer and more congruent scientific consensus.

## 2 Materials and methods

### 2.1 Literature search strategy

To identify relevant articles on how SARS-CoV-2 infection affects hypothyroidism and subclinical hypothyroidism, computer searches of the Willey Library, Embase, Web of Science, China National Knowledge Infrastructure (CNKI), Wanfang, and VIP databases were conducted. The search time is from December 2019 to August 2023. Keywords used in the search include “SARS-CoV-2”, ”hypothyroidism”, “thyroid dysfunction”, “subclinical hypothyroidism”, and corresponding Chinese terms.

### 2.2 Inclusion criteria

Design types included randomized controlled trials, case-control studies, or cohort studies written in English, regardless of sample size. The subjects were patients with hypothyroidism or subclinical hypothyroidism (serum thyroxine (T4) and triiodothyronine (T3) levels were normal, but serum thyroid stimulating hormone (TSH) levels were elevated). The experimental group was infected with SARS-CoV-2 (divided into mild and severe), and the control group was made up of uninfected individuals. The COVID-19 condition severity can be evaluated based on the following indices, including clinical symptoms, laboratory and radiographic abnormalities, hemodynamics and organ function (17). Mild cases refer to individuals with mild clinical symptoms but no shortness of breath or abnormal chest imaging. Severe cases refer to individuals with an oxygen saturation (SpO_2_) less than 93%, an oxygenation index (PaO2/FiO2) less than 300 mmHg, a respiratory rate greater than 30 breaths/min, or lung infiltrates greater than 50% of the total lung volume. The hypothyroidism patients infected with SARS-CoV-2 were further analyzed according to the severity of COVID-19. The outcome indices included in the study were TSH, TT3, FT3, TT4, and FT4.

### 2.3 Exclusion criteria

We excluded non-clinical controlled trials (e.g. reviews, guidelines, expert consensus), as well as those with repeated publication, missing data, or statistical errors. Also excluded were studies in which the outcome indices did not include TSH, TT3, FT3, TT4, and FT4.

### 2.4 Literature screening and data extraction

Preliminary screening was carried out independently by two researchers using the studies’ title and abstract. Both read the full texts of any articles that passed the initial screening to see if these should be included. The two researchers extracted information such as topic, author, publication year, research object, sample size, intervention methods, and important outcome indicators. If there were disagreements throughout the extraction process, an agreement was reached through discussion.

### 2.5 Literature quality evaluation

We used the Newcastle-Ottawa Scale (NOS) to rate the quality of each study that was included (14). The NOS scale is a three-part assessment instrument created especially for observational studies to assess study selection, comparison, and outcome. The scale assesses sample comparability, sample selection, and whether each study’s possible confounding variables are deemed adequate. Each article is eligible for a maximum of 9 points under the NOS scoring guidelines. Studies that receive a 7 or more are deemed to be of high literary quality, 4-6 are deemed to be of medium literary quality, and 3 or less are deemed to be of poor literary quality. Two independent reviewers rated each included studies, and when there were disagreements, they discussed them or sought the opinions of other reviewers.

### 2.6 Statistical analysis

The meta-analysis in this study was carried out employing R 4.2.1 software, using meta and metafor statistical packages. The mean difference value is frequently utilized for calculating the combined effect size in a meta-analysis. For continuous variables, we selected the Standardized Mean Difference (SMD) as the effect size and reported a 95% confidence interval (95% CI). If *I^2<50%* and *P>0.1*, the fixed effect model was applied. Otherwise, the random effect model was used, and the risk bias analysis method employed the Egger test. Statistics were judged significant at *P<0.05*.

## 3 Results

### 3.1 Characteristics of included studies

**Figure 1:**
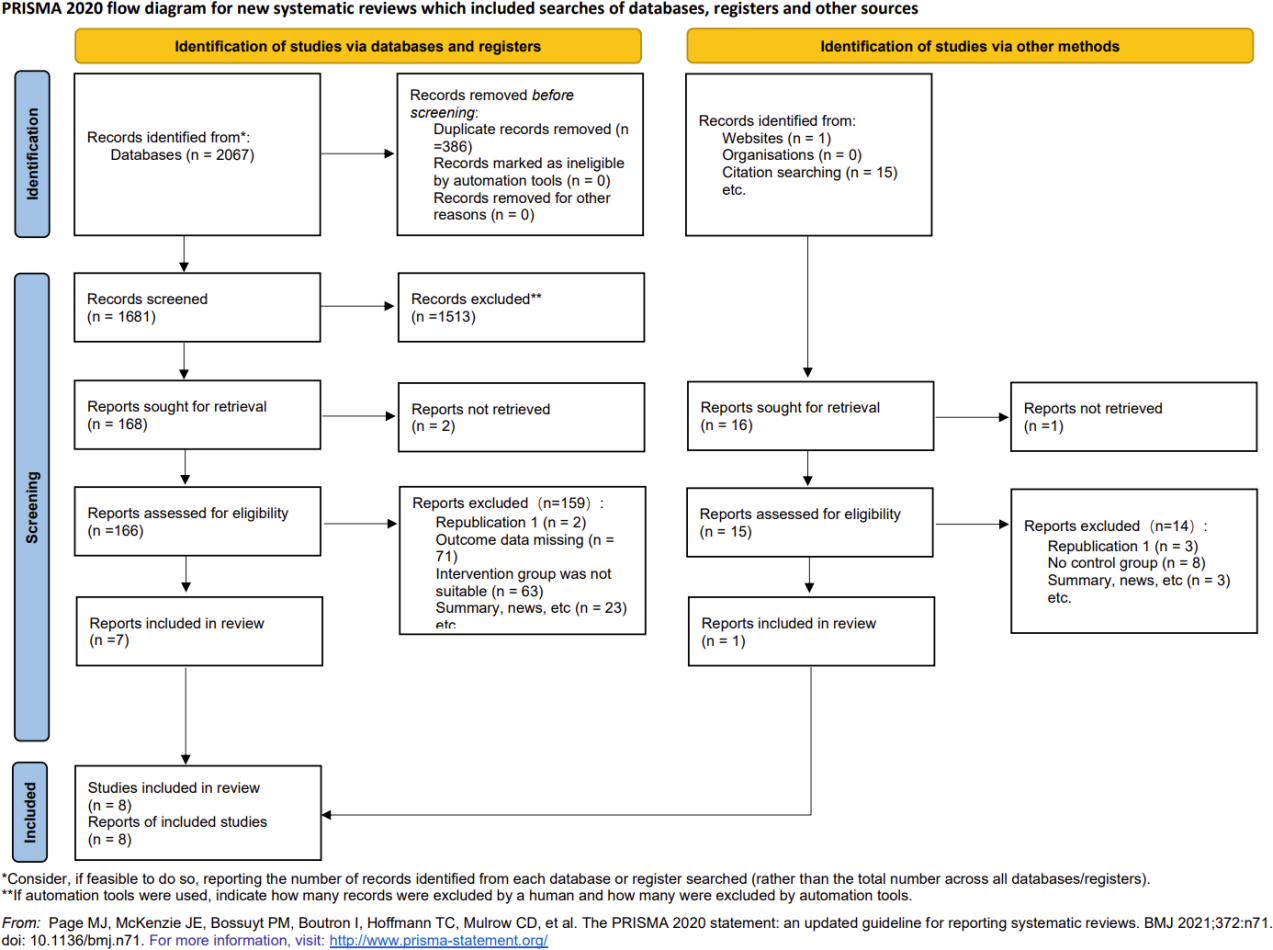
Flow diagram of the study selection process.

Overall, 2067 articles were retrieved from the database, 386 duplicate articles were excluded, 1513 articles with obvious inconformity were removed through the title and abstract reading, and the remaining 168 articles were downloaded. Seven of the articles were retained after final reading while one addition article was included through web search and literature tracking. Ultimately, a total of 8 articles were included (**Figure 1**), all of which were cohort or case-control studies involving participants from different nations, such as China, India, Turkey, Pakistan, Nigeria, and Bangladesh. The detailed information is shown in **Table 1**. The quality of articles is provided in **Table 2**. The majority of the studies included in this analysis received scores of 6 to 8 points according to the Newcastle-Ottawa Scale (NOS), indicating that they were of medium or high quality. After the quality assessment, no study was disqualified because of lack of reliability.

**Table 1:** Characteristics of the included studies.

Study	Country	Sample Size				Age(Year, mean ± SD)	Male(%)	Outcome
		COVID-19	Control	Severe	Mild			
Chen 2021 (10)	China	50	54	/	/	/	/	TSH、TT3、TT4
Dabas 2021 (18)	India	/	/	64	22	53.85 ± 19.54	63.70%	TSH
Dutta 2021 (19)	India	/	/	36	200	65(15~91)	42.50%	TSH、FT3、FT4
Gao 2021 (11)	China	/	/	66	34	62.6 ± 14.6	50.80%	TSH、FT3、FT4
Güven 2021 (20)	Turkey	/	/	125	125	65(46~83)	63%	TSH、FT3、FT4
Malik 2021 (13)	Pakistan	48	28	9	22	57.5 ± 16.6	59.30%	TSH、TT3、TT4
Okwor 2021 (16)	Nigeria	45	45	/	/	36.5 ± 10.6	26.70%	TSH、FT3、FT4
Razu 2022 (21)	Bangladesh	/	/	10	30	/	/	TSH、TT3、TT4

**Table 2:** Quality evaluation of the included studies.

Study	Case selection				Comparability	Exposure factor			Total
	1	2	3	4		A	B	C	
Chen 2021 (10)	1	1	1	1	1	1	1	1	8
Dabas 2021 (18)	1	1	1	0	2	1	1	0	7
Dutta 2021 (19)	1	0	1	1	1	1	1	1	7
Gao 2021 (11)	1	1	1	0	1	1	1	0	6
Güven 2021 (20)	1	0	1	1	2	1	1	0	7
Malik 2021 (13)	1	1	1	0	1	1	1	0	6
Okwor 2021 (16)	1	0	1	1	1	1	1	1	7
Razu 2022 (21)	1	1	1	1	1	1	1	0	7

### 3.2 Meta-analysis results

#### 3.2.1 The effect of SARS-COV-2 infection on hypothyroidism and subclinical hypothyroidism

##### 3.2.1.1 TSH

**Figure 2:**
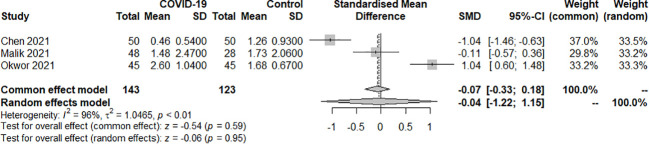
Forest plot of the effect of SARS-COV-2 infection on TSH in patients.

Three studies examined the TSH index of SARS-COV-2 infection in patients with hypothyroidism and subclinical hypothyroidism. A total of 266 samples were included, consisting of 143 patients and 123 controls. The random effect model well explained the heterogeneity test between studies, which had *P<0.01, I^2 = ^96%*. The findings revealed no difference in TSH levels between the COVID-19 group and the control group. [SMD=-0.04, 95%CI (-1.22, 1.15), *P=0.95*] (**Figure 2**).

##### 3.2.1.2 TT3

**Figure 3:**
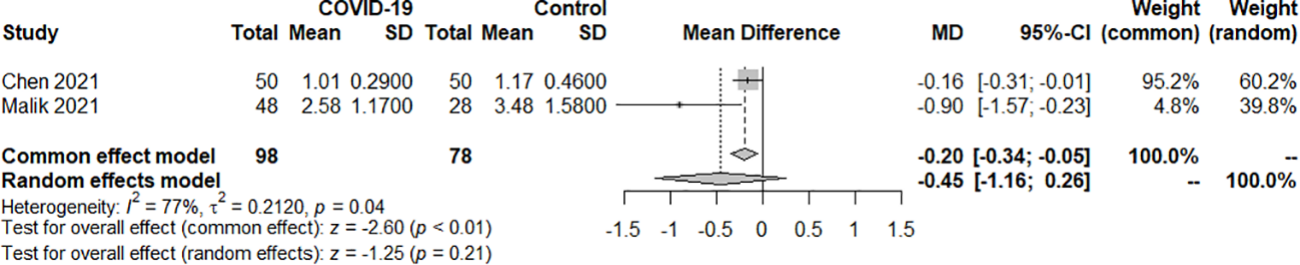
Forest plot of the effect of SARS-COV-2 infection on TT3 in patients.

Two studies examined the TT3 index of SARS-COV-2 infection in patients with hypothyroidism and subclinical hypothyroidism. A total of 180 samples were included, consisting of 90 patients and 90 controls. The random effect model well explained the heterogeneity test between studies, which had *P=0.04, I^2 = ^77%.* The findings revealed no difference in TT3 levels between the COVID-19 group and the control group. [SMD=-0.45, 95%CI (-1.16,0.26), *P=0.21*] (**Figure 3**).

##### 3.2.1.3 TT4

**Figure 4:**
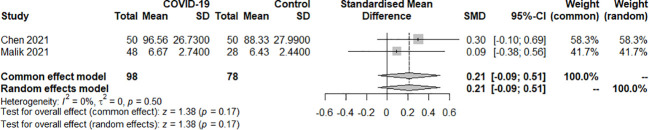
Forest plot of the effect of SARS-COV-2 infection on TT4 in patients.

Two studies examined the TT4 index of SARS-COV-2 infection in patients with hypothyroidism and subclinical hypothyroidism. A total of 176 samples were included, consisting of 98 patients and 78 controls. The fixed effect model well explained the heterogeneity test between studies, which had *P=0.50*, *I^2 = ^0%.* The findings revealed no difference in TT4 levels between the COVID-19 group and the control group. [SMD=0.21, 95%CI (-0.09,0.51), *P=0.17*] (**Figure 4**).

#### 3.2.2 The effect of COVID-19 severity on hypothyroidism and subclinical hypothyroidism

##### 3.2.2.1 TSH

**Figure 5:**
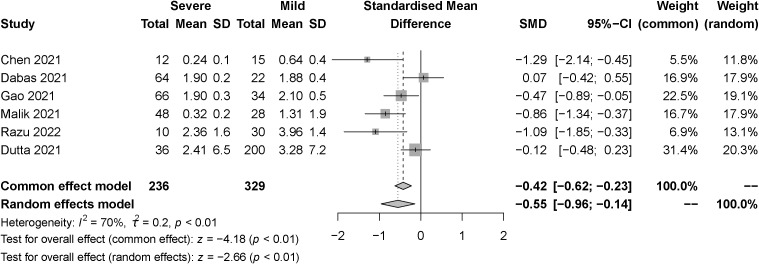
Forest plot of the effect of the severity of SARS-COV-2 infection symptoms on the patient’s TSH.

The effect of the severity of SARS-COV-2 infection on TSH markers in individuals with hypothyroidism and subclinical hypothyroidism was assessed in six studies. A total of 565 samples were included, consisting of 236 severe patients and 329 mild patients. The random effect model explained the heterogeneity test between studies, which was *P<0.01*, *I^2 = ^70%*. The findings indicated that TSH was significantly lower in the severe than the mild group. [SMD=-0.55, 95%CI (-0.96, -0.14), *P<0.01*] (**Figure 5**).

##### 3.2.2.2 FT3 and TT3

**Figure 6:**
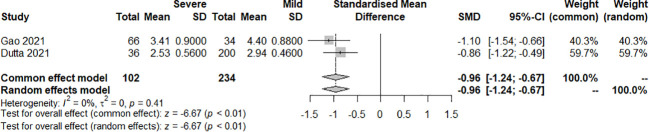
Forest plot of the effect of the severity of SARS-COV-2 infection symptoms on the patient’s FT3.

**Figure 7:**
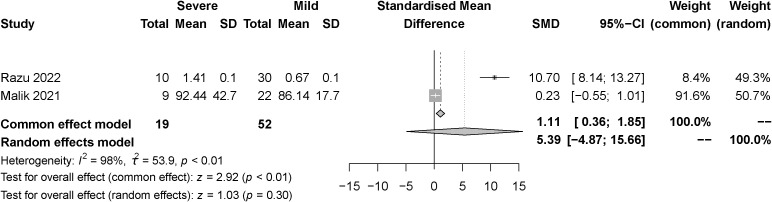
Forest plot of the effect of the severity of SARS-COV-2 infection symptoms on the patient’s TT3.

The effect of the severity of SARS-COV-2 infection on FT3 and TT3 markers in individuals with hypothyroidism and subclinical hypothyroidism was assessed in two studies. The random effect model and fixed effect model explained the heterogeneity test results between trials, which were *P<0.01*, *I^2 = ^98%* and *P=0.41*, *I^2 = ^0%*, respectively. According to the findings, FT3 was significantly lower in the severe than the mild group, [SMD=-0.96, 95%CI (-1.24,-0.67), *P<0.01*] (**Figure 6**) and TT3 showed no difference. [SMD=5.39, 95%CI (-4.87, 15.66), *P=0.03*] (**Figure 7**).

##### 3.2.2.3 FT4 and TT4

**Figure 8:**
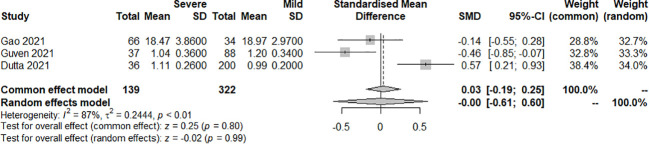
Forest plot of the effect of the severity of SARS-COV-2 infection symptoms on the patient’s FT4.

**Figure 9:**
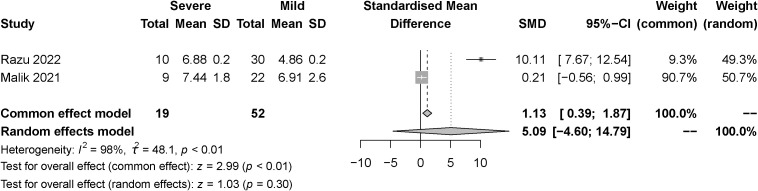
Forest plot of the effect of the severity of SARS-COV-2 infection symptoms on the patient’s TT4.

The effect of the severity of SARS-COV-2 infection on FT4 and TT4 markers in individuals with hypothyroidism and subclinical hypothyroidism was assessed in three and two studies respectively. The random effect model explained the heterogeneity test results between trials, which were *P<0.01*, *I^2 = ^87%* and *P<0.01*, *I^2 = ^98%*, respectively. Both studies were described by random effects model. The results indicated no difference between severe and mild groups in FT4. [SMD=-0.00, 95%CI (-0.61, 0.60), P=0.99] (**Figure 8**) Similarly, there was no difference between groups in TT4. [SMD=5.09, 95%CI (-4.60, 14.79), P=0.30] (**Figure 9**).

### 3.3 Risk of bias assessment

We performed a statistical analysis using the Egger test to determine the degree of bias in the included studies. The findings revealed all indicators had P values higher than 0.05, signifying no bias.

## 4 Discussion

The global outbreak of SARS-CoV-2 has attracted considerable attention, especially given its potential effects on multiple organs, including the thyroid gland (12, 13). Hypothyroidism and subclinical hypothyroidism are two high-risk endocrine conditions, each presenting with a range of clinical manifestations, symptoms, and biochemical changes. The thyroid gland can be susceptible to a wide range of internal and external factors, which can cause it to behave abnormally in some situations, such as systemic diseases or viral infections (22–24). However, the exact effect of SARS-CoV-2 on thyroid function remains a topic of interest. Therefore, a comprehensive meta-analysis is essential to systematically assess the potential impact of SARS-CoV-2 on thyroid function and provide guidance for future research and treatment.

In this meta-analysis, we systematically examined the effects of SARS-CoV-2 infection on thyroid function. Our overall findings showed that TSH, TT3, and TT4 levels were not significantly affected by SARS-CoV-2 infection, thus aligning with the current popular hypothesis that SARS-CoV-2 infection does not directly induce significant changes in thyroid function (15). However, our findings indicate several relevant effects when we investigated the impact of the severity of COVID-19. Specifically, individuals with severe COVID-19 showed disturbed thyroid function, reflecting lower TSH and FT3 levels compared with those with mild COVID-19.

A number of explanations are plausible for the above observations. Individuals with severe COVID-19 frequently experience a strong systemic inflammatory response, which may have an effect on how thyroid hormones are transformed and released (25). For instance, inflammation may cause cystosulfate deiodinase (DIO2) to function less efficiently, which may influence the conversion of T4 to T3 (26). Also, the virus might directly or indirectly invade thyroid tissues, causing inflammation and cellular damage (27). Furthermore, a variety of pharmacological therapies are frequently administered to critically sick patients, some of which may affect thyroid function (25, 28). For instance, systemic corticosteroids are recommended for use in severe COVID-19 patients while inhibiting serum TSH levels (25). It is worth noting that while these alterations may suggest thyroid dysfunction, it remains uncertain if they correlate directly with clinical outcomes like mortality or hospitalization duration (29, 30). Future studies should further explore these potential mechanisms and their effects on patients’ clinical prognoses.

This meta-analysis has several significant advantages. First, we adopted a systematic methodology and rigorous data analysis process to ensure the accuracy and reliability of the research results. Second, our study is the first systematic assessment of the association between SARS-CoV-2 infection and thyroid function changes, providing new insights into this field. In addition, our study covers data from multiple countries and regions, increasing the breadth and diversity of research. These characteristics make this study have a high reference value in assessing the impact of SARS-CoV-2 on thyroid function.

However, our research also has some limitations. Although we evaluated the quality of the included studies using the NOS scale, there may still be some confounding factors because of the various data sources. Despite our meta-analysis encompassing multiple studies, the sample size remains relatively limited, potentially affecting our statistical robustness and the uniformity of our findings.

## 5 Conclusion

In conclusion, while SARS-CoV-2 infection itself may not directly induce hypothyroidism, the severity of COVID-19 may contribute to thyroid dysfunction. Specifically, TSH and FT3 levels were significantly lower in individuals with severe COVID-19 than those with mild COVID-19. It is important to explore these findings further in order to understand the potential mechanisms of thyroid dysfunction in the outcome of COVID-19.

## Data availability statement

The original contributions presented in the study are included in the article/supplementary material. Further inquiries can be directed to the corresponding author.

## Author contributions

JW: Writing – original draft, Writing – review & editing, Data curation, Validation. FZ: Writing – review & editing, Funding acquisition, Project administration, Supervision.
